# Chilaiditi Sign: Rare Incidental Finding on Chest Radiograph

**DOI:** 10.5811/westjem.2015.10.28653

**Published:** 2015-12-11

**Authors:** Krystal Garcia, John Ashurst

**Affiliations:** * Edward Via College of Osteopathic Medicine, Carolinas Campus, Spartansburg, South Carolina; † Duke Lifepoint Memorial Medical Center, Department of Emergency Medicine, Johnstown, Pennsylvania

A 68-year-old male with a history of prostate cancer presented with a two-day history of fever and left flank pain. Vital signs included a temperature of 39.4 degrees Celsius with 93% oxygen saturation and heart rate of 112 beats per minute. An upright chest radiograph showed concern for free intraperitoneal air (Figure) with a white blood cell count of 17.3. A computed tomography of the abdomen and pelvis revealed a Chilaiditi sign with pyelonephritis, which was confirmed on urinalysis. He was admitted for intravenous antibiotics.

## DISCUSSION

Chilaiditi sign, also called pseudopneumoperitoneum, is named after the Greek radiologist, Dmitri Chilaiditi, who first described it in 1910.^1^ It is an interposition of bowels between the liver and right diaphragm and appears as free air on chest radiograph.^2^,^3^ This sign is found in <0.3% of the population with highest incidence in elderly males.^2^ To diagnosis Chilaiditi sign, the following criteria must be met: (1) right hemidiaphragm must be elevated above liver by intestine, (2) bowel must be distended by air, (3) and the superior margin of the liver must be depressed below the level of the left hemidiaphragm.

If symptomatic, this is referred to as Chilaiditi syndrome, which can manifest as abdominal or cardiac symptoms with self-resolution or chronicity.^1^,^2^ Usually only conservative treatment is required for patients with Chilaiditi syndrome, but surgery may be needed for severe cases.^1^ The emergency physician should be aware of this condition as a potential mimicker of intraperitoneal free air on chest radiograph.

## Figures and Tables

**Figure:**
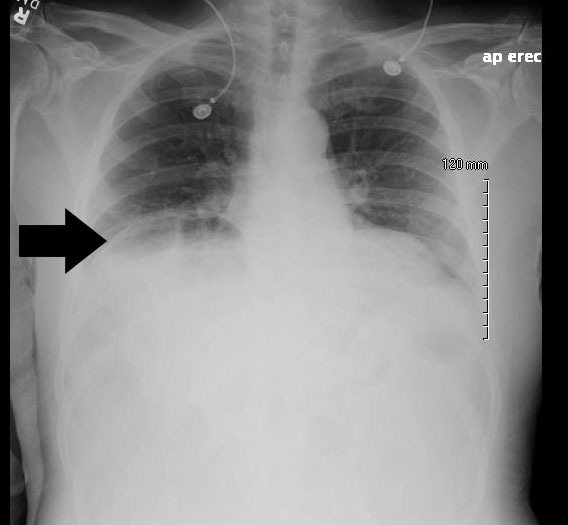
Upright chest radiograph demonstrating Chilaiditi sign (pseudopneumoperitoneum) mimicking apparent free intraperitoneal air under the right hemidiaphragm.
